# Performance of Warm Mix Asphalt with Polymer Modified RAP Using Recycled Engine Oil and SBS Binder Modification

**DOI:** 10.3390/polym18010044

**Published:** 2025-12-23

**Authors:** Byung-Sik Ohm, Tri Ho Minh Le

**Affiliations:** 1 Department of Highway & Transportation Research, Korea Institute of Civil Engineering and Building Technology, 283 Goyangdae-Ro, Ilsanseo-Gu, Goyang-si 10223, Gyeonggi-Do, Republic of Korea; 2 Faculty of Civil Engineering, Nguyen Tat Thanh University, 300A Nguyen Tat Thanh Street, District 4, Ho Chi Minh City 70000, Vietnam

**Keywords:** warm-mix asphalt, polymer-modified RAP, recycled engine oil, SBS polymer, rheology, mechanical performance

## Abstract

The growing use of reclaimed asphalt pavement (RAP) in warm-mix asphalt (WMA) presents significant challenges when RAP originates from aged polymer-modified binder (PMB) pavements, where severe oxidation and polymer degradation lead to excessive stiffness and poor cracking resistance. This study presents a multi-scale evaluation of a hybrid modification strategy combining recycled engine oil waste (REOW, 3 wt.%) and styrene–butadiene–styrene (SBS, 1–4 wt.%) to restore aged PMB-containing RAP systems under controlled binder conditions. Three binders (control, REOW-modified, and REOW–SBS hybrid) were prepared using a fixed 70/30 virgin-to-RAP binder blend and characterized through rheological analysis, and multiple stress creep recovery (MSCR). The findings show that REOW softened the binder but reduced elastic recovery, whereas SBS modification restored elastic response. Corresponding WMA mixtures with 30 wt.% RAP and 5.0 wt.% total binder content were evaluated for moisture damage, raveling, rutting, and cracking resistance. At the mixture scale, the hybrid system achieved a TSR of 83%, reduced Hamburg rut depth by ~20%, and increased SCB fracture energy by ~30% compared with the control. These findings demonstrate that combined rejuvenation–reinforcement effectively re-mobilizes aged PMB chemistry and restores polymer elasticity, enabling high-performance WMA production with RAP derived from polymer-modified pavements.

## 1. Introduction

The increased demand for durable, cost-effective, and sustainable pavement materials has intensified interest in incorporating RAP into both hot-mix and WMA technologies. RAP, however, contains aged binder that has undergone oxidative hardening, polymer degradation, and volatilization, resulting in significantly higher stiffness and reduced ductility compared with virgin asphalt [1]. Although RAP offers substantial environmental and economic benefits, high RAP contents often reduce mixture flexibility and increase cracking risk. This issue becomes more severe when the original pavement used polymer-modified binders (PMBs), where aging induces SBS chain scission and loss of elastic recovery, producing highly stiff, low-recovery RAP binders that are difficult to reintegrate without additional modification.

WMA technologies have further encouraged RAP utilization by lowering production temperatures and reducing thermal aging [2]. Yet integrating high RAP contents into WMA remains challenging: reduced temperatures limit binder mobilization, slow blending between virgin and aged binder phases, and may increase moisture susceptibility. Rejuvenators have thus become essential for RAP-rich WMA systems. REOW has gained attention due to its high content of light hydrocarbon fractions that reduce asphaltene agglomeration and improve binder fluidity; however, it does not supply resin components and therefore cannot fully substitute for the complete maltene system. Instead, REOW functions by partially restoring the colloidal balance of aged binders through plasticization rather than full maltene replenishment.

Recently, waste engine oil has also been increasingly explored as a secondary raw material for bituminous binders due to its high content of light maltene-like fractions, which compensate for the loss of aromatics during aging and help restore the colloidal balance of hardened binders [3]. Korchak et al. [4] demonstrated that used mineral motor oils can be effectively regenerated through an integrated thermo-oxidative treatment, vacuum distillation, and urea purification process, producing regenerated oils and residues suitable for bitumen-related applications. This provides a technical basis for considering regenerated waste engine oils as plasticizing or rejuvenating agents in asphalt binder modification [5]. In parallel, modern bitumen modification relies on several additive groups: thermoplastic polymers (e.g., LDPE [6], EVA [7]), thermoelastoplastics such as SBS that form elastic networks [8], plasticizers that improve workability, adhesion promoters that enhance aggregate–binder bonding, and secondary raw materials that partially substitute fresh bitumen. Within this classification, REOW functions primarily as a rejuvenator/plasticizing secondary raw material, whereas SBS is a thermoelastoplastic modifier that increases elasticity and high-temperature performance.

Polymer modifiers such as SBS are widely used to rebuild elasticity and improve high-temperature performance. Recent studies have explored warm-mix technologies with rubber or polymer additives (Hu et al. [9]; Yu et al. [10]), multi-scale adhesion mechanisms in recycled SBS binders (Li et al. [11]), rubber and fiber reinforcement (Jin et al. [12]), and polymer-modified mixture performance (Alsolieman et al. [13]; Safaeldeen et al. [14]). Parallel research has investigated rejuvenation strategies using REOW/REOB-type oils (Porto et al. [15]; Li et al. [16]; Shi et al. [17]) and the combined use of oil-based rejuvenators with polymers (Li et al. [18]). RAP-oriented mixture studies (Hoy et al. [19]; Lee and Le [20]; Lee et al. [21]) and comprehensive reviews on RAP blending behavior (Xing et al. [22]) further highlight the complexities associated with recycled PMB binders.

Another important knowledge gap concerns the translation of binder-level improvements to mixture-level behavior, particularly for high-RAP warm-mix asphalt produced from polymer-modified pavements. Although rejuvenators can reduce stiffness and polymers such as SBS can enhance elasticity at the binder scale, their combined effects on mixture rutting, raveling, moisture damage, and cracking are not always straightforward. Field-aged polymer-modified binders commonly exhibit oxidation-induced hardening and degradation of the SBS network, making re-modification with SBS alone insufficient to restore viscoelastic balance. In such systems, a plasticizing component is required to re-mobilize the aged binder phase prior to effective polymer reinforcement.

High RAP contents further introduce complexities related to aggregate structure, binder film thickness, and moisture susceptibility under warm-mix asphalt conditions. Comprehensive multi-scale studies linking binder rheology (viscosity, master curves, MSCR recovery), aging behavior (RTFO, PAV), and mixture performance (ITS/TSR, Cantabro, Hamburg wheel tracking, Overlay Test, and SCB fracture energy) remain scarce, particularly for RAP derived from aged polymer-modified pavements. Against this background, this study proposes a hybrid REOW–SBS modification strategy and provides a systematic multi-scale evaluation of its effectiveness in restoring binder functionality and improving mixture performance, thereby addressing both scientific and practical challenges associated with high-RAP warm-mix asphalt.

Therefore, the objective of this study is to investigate the feasibility of combining REOW and SBS polymer to rehabilitate a field-aged RAP binder and to evaluate the resulting binder- and mixture-level performance of warm-mix asphalt containing 30 wt.% RAP at the mixture scale. In this manuscript, the term bitumen refers exclusively to the asphalt binder material, while asphalt concrete denotes the composite mixture of binder and mineral aggregates; these terms are used consistently to avoid ambiguity. To achieve this objective, the RAP binder was first extracted and recovered from reclaimed wearing-course materials and used for binder-level characterization and controlled blending studies. The recovered RAP binder was blended with virgin 60/70 binder to prepare base binders, which were then modified using recycled engine oil waste (3 wt.%) and SBS polymer (1–4 wt.%) to examine rejuvenation and polymer-reinforcement mechanisms under controlled binder conditions. Subsequently, warm-mix asphalt concretes incorporating 30 wt.% RAP aggregate were produced. The RAP asphalt content measured from extraction was used to determine the binder contribution from RAP, while virgin binder (modified according to the selected binder formulation) was added by difference to achieve a fixed total binder content. Mixture performance was evaluated through ITS/TSR, Cantabro loss, Hamburg wheel tracking, Overlay Test fatigue response, and SCB fracture energy. This integrated framework enables direct linkage between binder rheological behavior and asphalt concrete performance, providing insight into the feasibility of REOW–SBS hybrid modification for RAP-rich warm-mix asphalt systems.

## 2. Materials and Methods

### 2.1. Materials

#### 2.1.1. Binder

Two types of bitumen were used in this study: a conventional 60/70 penetration-grade virgin binder and a polymer-modified RAP binder recovered from reclaimed asphalt pavement. The virgin 60/70 binder is a standard paving grade commonly used in Vietnamese dense-graded asphalt mixtures, with a penetration of 60–70 dmm, a softening point of approximately 46–50 °C, and a rotational viscosity of about 350–450 mPa·s at 135 °C.

In addition to the raw materials, a “Control Binder” (denoted as B0) was established to serve as the reference baseline. This binder was produced by blending 70 wt.% virgin 60/70 bitumen with 30 wt.% recovered RAP binder, simulating the combined binder phase of the reference warm-mix asphalt mixture prior to the addition of any rejuvenators or polymer modifiers. Its physical and rheological properties were characterized and are listed in Table 1 alongside the base components.

**Figure 1:**
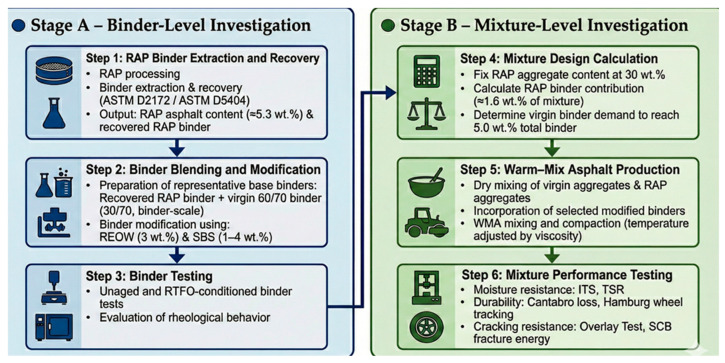
Experimental workflow for RAP-containing warm-mix asphalt.

In this study, the RAP binder was first extracted and recovered from reclaimed wearing-course materials in accordance with ASTM D2172 [23] and ASTM D5404 [24] to enable binder-level testing and controlled binder blending investigations. The reclaimed pavement originated from an expressway surface layer and was originally constructed using a polymer-modified binder (PMB). The extraction process provided the RAP asphalt content (≈5.3 wt.%), which served as a key input for both the binder study and the subsequent mixture design. The general work flow is presented in Figure 1.

At the binder scale, the recovered RAP binder was blended with virgin 60/70 binder to prepare representative base binders for rheological evaluation and modification studies. REOW and SBS polymer were incorporated into these blended binders to investigate rejuvenation and polymer reinforcement mechanisms under controlled binder conditions (Section 3.1).

Following the binder-level investigation, the study proceeded to mixture-level validation. RAP was incorporated into warm-mix asphalt mixtures at 30 wt.% of total aggregate mass. Based on the measured RAP asphalt content, the RAP binder contribution to the mixture was calculated (≈1.6 wt.% of mixture mass). The total binder content was fixed at 5.0 wt.%, and virgin binder—modified according to the selected binder formulations—was added by difference to achieve the target binder content. Thus, binder proportions in the mixtures were determined by RAP asphalt content and volumetric mix design, rather than by predefined binder blending ratios.

To assess the suitability of the virgin binder and the recovered RAP binder for asphalt mixture production, their fundamental physical and rheological properties were first characterized and compared against typical requirements for paving-grade bitumen. This preliminary evaluation is intended to establish whether each material can be used directly as a binder or requires further treatment. Table 1 summarizes the penetration, softening point, ductility, viscosity, and key rheological parameters of the virgin 60/70 bitumen and the recovered polymer-modified binder from RAP, providing a basis for evaluating aging susceptibility and standalone binder usability prior to modification. Virgin paving-grade 60/70 binder meeting TCVN 7493 [25]/ASTM D946 [26] is intended for direct use in HMA production; recovered RAP binder generally requires blending/rejuvenation to meet paving-grade or performance specifications.

**Table 1:** General properties of virgin binder and recovered RAP binder.

Property	Typical Requirement	Virgin 60/70 Bitumen in This Research	Current RAP Bitumen (Recovered PMB)	Control Binder (B0)	Assessment	Test Method
Penetration (25 °C, 100 g, 5 s)	60–70 dmm	65 dmm	20 dmm	41 dmm	RAP binder far outside acceptable range	ASTM D5 [27]
Softening point (Ring and Ball)	≥46 °C	48 °C	65 °C	58.5 °C	RAP binder excessively hardened	ASTM D36 [28]
Ductility (25 °C)	≥75–80 cm	>80 cm	8 cm	-	Severe embrittlement of RAP binder	ASTM D113 [29]
Rotational viscosity (135 °C)	≤3.0 Pa·s	0.42 Pa·s	1.75 Pa·s	0.685 Pa·s	Poor workability of RAP binder	ASTM D4402 [30]
G*/sinδ (64 °C, unaged)	≤2.2 kPa (Superpave guideline)	1.2 kPa	8.0 kPa	4.8 kPa	RAP binder overly stiff	ASTM D7175 [31]
MSCR Jnr_3.2_ (64 °C, unaged)	≤2.0 kPa^−1^ (traffic dependent)	2.3 kPa^−1^	0.6 kPa^−1^	1.34 kPa^−1^	Limited stress relaxation	ASTM D7405 [32]
Standalone binder usability	Required	Suitable	Not suitable without rejuvenation		-	

* Typical ranges derived from penetration-grade and Superpave practice; values are used for qualitative comparison rather than formal certification.

#### 2.1.2. Aggregate Mix

Crushed basalt aggregates sourced from a local quarry in southern Vietnam were used for all mixture preparations. Basalt is the predominant aggregate type employed in dense-graded wearing courses due to its high angularity, excellent interlock potential, and superior mechanical strength compared with sedimentary aggregates. The aggregates were oven-dried and separated into individual size fractions (19 mm, 13.2 mm, 9.5 mm, 4.75 mm, 2.36 mm, 0.425 mm, and filler) according to ASTM C136 [33] to prepare the specified 13 mm NMAS dense-graded gradation.

Key physical properties of the coarse and fine aggregates used in this study are summarized in Table 2. Aggregates consisted of crushed granite and natural sand blends commonly used in WMA–RAP applications.

**Figure 2:**
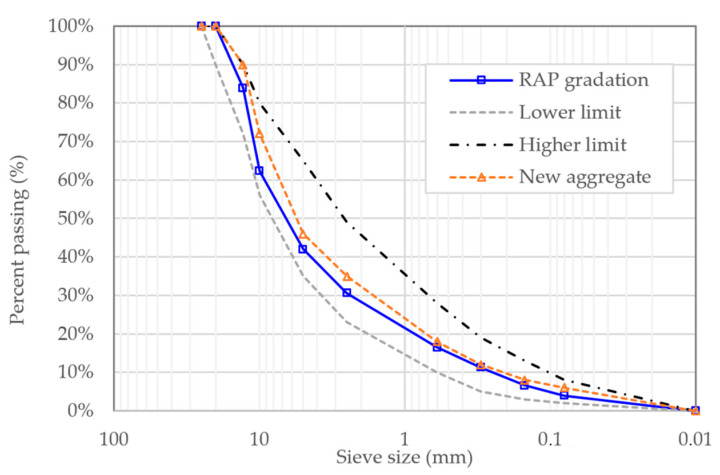
Sieve size analysis of the aggregate used in this research.

Aggregate absorption was measured following ASTM C127 [34] and C128 [35], yielding values of 0.9–1.1% for coarse aggregates and 1.2–1.4% for fine aggregates. These absorption characteristics were incorporated into binder content calculations to ensure accurate volumetric control. The selected aggregates provided a high-quality skeleton structure necessary for the evaluation of binder modification effects in mixtures containing 30% RAP. The sieve size of aggregate used in this research is presented in Figure 2 while general physical properties of the aggregates are presented in Table 2.

**Table 2:** Physical properties of basalt aggregates.

Property	Coarse Aggregate	Fine Aggregate	Test Method
Specific gravity (SSD)	2.83	2.80	ASTM C127/C128 [34]
Water absorption (%)	1.0	1.3	ASTM C127/C128 [34]
Los Angeles abrasion (%)	21%	-	ASTM C131 [36]
Flakiness index (%)	18%	-	BS 812 [37]
Aggregate type	Crushed basalt	Crushed basalt	-

RAP aggregates, after binder extraction, were characterized separately to determine their independent physical properties. The extracted RAP aggregates demonstrated slightly higher absorption (1.5–1.8%) compared with virgin basalt, attributed to residual binder coating and microcracking generated during milling. The Los Angeles abrasion ranged between 18–20%, still within acceptable limits due to the high-quality basalt parent rock. RAP gradation was screened and adjusted to match the target job-mix formula, ensuring the RAP fraction contributed appropriately to the mixture’s coarse and fine aggregate skeleton.

RAP was incorporated at 30 wt.% by total aggregate mass, which corresponds to approximately 30 wt.% RAP binder replacement in the blended binder formulation. This RAP content reflects common practice in warm-mix RAP applications and provides a meaningful level of binder aging influence for evaluating the effects of REOW and SBS modification.

The general physical and binder recovery properties of the RAP material are summarized in Table 3.

**Table 3:** General properties of RAP materials.

Property	RAP Aggregate	Test Method
Maximum size	19 mm	ASTM C136 [33]
Asphalt content (%)	5.2–5.6%	ASTM D2172 [23]
Moisture content (%)	<1%	ASTM D1461 [38]
Specific gravity (SSD)	2.70–2.74	ASTM C127/C128 [34]
Water absorption (%)	1.5–1.8%	ASTM C127/C128 [34]
Los Angeles abrasion (%)	18–20%	ASTM C131 [36]

#### 2.1.3. Additives

Two additives were used to modify the blended binder systems: REOW as a rejuvenating agent and SBS as a polymer modifier. These additives were selected to evaluate the combined restoration and reinforcement mechanisms required for warm-mix RAP applications with a high proportion of aged polymer-modified binder.

In accordance with recent classifications of bitumen additives, waste engine oil derivatives are considered plasticizers due to their ability to increase binder fluidity and reduce stiffness by replenishing maltene fractions, as discussed in recent studies [39]. According to Prysiazhnyi et al. (2024) [39], plasticizing additives for road bitumen are defined as materials that increase penetration and ductility while only marginally affecting the softening point, a functional behavior that justifies classifying recycled engine oil waste as a plasticizer rather than a reactive modifier.

REOW is an aromatic-rich, low-viscosity petroleum-derived plasticizer-type rejuvenator, consistent with recent classifications of waste engine oil derivatives, and is used to restore aged binder by replenishing lost maltenes and reducing asphaltene agglomeration [15]. Its composition typically includes light maltene fractions, dispersants, and residual antioxidants, which facilitate the restoration of aged RAP binder by replenishing lost aromatics and reducing asphaltene agglomeration. The general properties of REOW is presented in Table 4.

**Table 4:** Properties of REOW rejuvenator and SBS polymer.

Property	REOW	SBS Polymer	Test Method
Appearance	Dark brown, low-viscosity liquid	Light-colored granules	Visual
Viscosity (135 °C)	<100 mPa·s	-	ASTM D445 [40]
Flash point (°C)	>220 °C	-	ASTM D92 [41]
Specific gravity	0.88–0.92	0.94–0.98	ASTM D70 [42]
Ash content (%)	<0.5%	-	ASTM D482 [43]
Styrene content (%)	-	~30%	Manufacturer data
Polymer type	-	Linear SBS (tri-block)	-
Melt flow index	-	0.8–1.2 g/10 min	ASTM D1238 [44]

SBS polymer was used to strengthen the modified binder system and to offset the high-temperature softening effect of REOW. The SBS employed in this study was a linear triblock copolymer with a styrene content of approximately 30% and a melt flow index of 0.8–1.2 g/10 min. This polymer type is widely used in PMB applications due to its ability to form an elastic three-dimensional network that enhances rutting resistance, elastic recovery, and strain-tolerance under repeated loading.

For hybrid modification, REOW was first blended into the virgin RAP binder mixture to ensure uniform diffusion and softening of the aged binder components. SBS was then added under high-shear mixing to achieve complete polymer swelling and dispersion. This sequential approach ensured both effective rejuvenation of the RAP binder fraction and proper network formation of the SBS polymer. The general characteristics of REOW and SBS used in this study are summarized in Table 4**.**

### 2.2. Binder Preparation and Blending Procedure

All binders were prepared by blending 30 wt.% recovered RAP binder with 70 wt.% virgin 60/70 binder, followed by the incorporation of REOW and SBS according to the target modification levels as shown in Table 5. The blending procedure was designed to ensure proper softening of the aged RAP binder, complete diffusion of rejuvenator, and full polymer development for the SBS-modified systems. The general workflow followed ASTM D7173 [45] recommendations for binder conditioning and modification.

The virgin 60/70 binder was heated to 150 ± 5 °C, while the recovered RAP binder was preheated to 160 °C to ensure adequate fluidity. The two binders were blended for 20 min at 700 rpm under controlled temperature to obtain a homogeneous base binder.

For REOW-modified binders (B1 and B4), the rejuvenator was added at 135–140 °C and mixed for 20 min at 500–700 rpm to promote effective diffusion into the aged RAP binder. For SBS-modified binders (B4), SBS pellets were subsequently introduced at 175–180 °C and mixed under high shear (3000–4000 rpm) for 60 min to ensure complete polymer swelling and network formation. After modification, SBS-containing binders were conditioned for an additional 30 min at 160 °C to stabilize the polymer structure.

Binder characterization was conducted on unaged and RTFO-conditioned samples. RTFO aging was applied in accordance with ASTM D2872 [46] solely as a short-term conditioning step to simulate the oxidative and volatilization effects associated with warm-mix asphalt production and RAP incorporation. The purpose of RTFO conditioning was not to evaluate binder aging resistance, but to ensure that rheological testing was performed under conditions representative of mixture production.

### 2.3. Binder Testing Methods

A comprehensive binder testing program was conducted to evaluate the effects of RAP, REOW, and SBS modification on binder rheology and to establish the performance characteristics needed for subsequent mixture-level assessment. All testing protocols followed ASTM and AASHTO standards to ensure reproducibility and comparability with existing literature.

Penetration (ASTM D5 [27]), softening point (ASTM D36 [47]), ductility (ASTM D113 [29]), and rotational viscosity (ASTM D4402 [30]) were measured to establish the baseline physical characteristics of the virgin binder, recovered RAP binder, and REOW–SBS modified binders, and to screen the appropriate REOW dosage (1–4 wt.%) prior to selecting the final formulation. Short-term and long-term aging were performed using the RTFO (ASTM D2872 [46]) at 163 °C and the PAV (ASTM D6521 [48]) at 100 °C for 20 h, ensuring that the binders reflected realistic aging conditions associated with warm-mix production and RAP incorporation.

**Figure 3:**
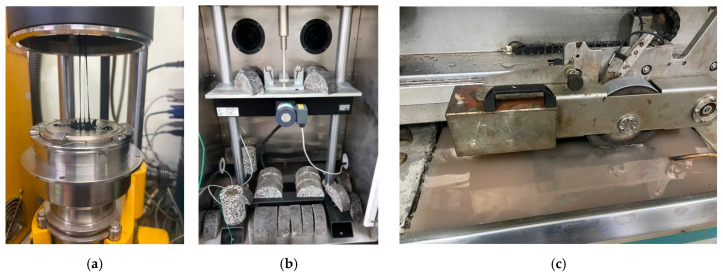
(**a**) Dynamic Shear Rheometer; (**b**) SCB testing; (**c**) HWT testing.

Rheological characterization was conducted using a Dynamic Shear Rheometer (ASTM D7175 [31]) (Figure 3a), including temperature sweep tests (46–82 °C) to determine complex shear modulus (G*). Frequency sweep tests (0.1–100 rad/s) were further used to construct master curves using time–temperature superposition, providing detailed insight into binder viscoelasticity, stiffness evolution, and temperature susceptibility across modification levels.

MSCR tests were performed in accordance with ASTM D7405 [32] to evaluate high-temperature deformation resistance and elastic recovery of the modified binders. Tests were conducted at 3.2 kPa and 0.1 kPa stress levels, capturing both stress sensitivity and polymeric recovery behavior.

### 2.4. Warm Asphalt Mixture Preparation

WMA mixtures were produced using REOW as the warm-mix additive to enable reduced production temperatures while incorporating a high RAP content. All mixtures followed a 13 mm NMAS dense-graded gradation and contained 30 wt.% RAP aggregate by total mixture mass.

The average asphalt content of RAP was determined by extraction to be approximately 5.3 wt.%, resulting in a RAP binder contribution of 1.6 wt.% of the mixture. The total binder content was fixed at 5.0 wt.%, and virgin 60/70 binder (with or without REOW and SBS modification) was added by difference to achieve the target binder content. No predefined RAP binder–virgin binder blending ratio was imposed.

Three main mixture types were prepared: M0 using the control binder (B0), M1 incorporating REOW-modified binder (B1), and M4 incorporating the hybrid REOW–SBS modified binder (B4). The mixture preparation procedure was kept identical for all mixtures, with adjustments applied only to mixing and compaction temperatures to account for viscosity reductions induced by the WMA additive.

Virgin aggregates were oven-dried at 110 ± 5 °C and preheated to their designated mixing temperature. RAP materials were dried at 60–70 °C to prevent additional oxidative aging while ensuring sufficient moisture removal. Virgin and RAP aggregates were then combined in their specified proportions and dry-mixed to achieve temperature uniformity prior to binder addition. The prepared binder was introduced gradually under continuous mechanical mixing to ensure uniform coating of both virgin and RAP aggregates. Mixing temperatures were set at 160–165 °C for the control mixture and reduced to 145–150 °C for REOW-containing mixtures, based on binder viscosity results reported in Section 2.3.

Compaction was carried out using a Superpave gyratory compactor at the corresponding compaction temperatures. The control mixture (M0) was compacted at 150–155 °C, whereas REOW-containing mixtures (M1 and M4) were compacted at 135–140 °C due to enhanced binder mobility. All mixtures were compacted to N_design = 75 gyrations, targeting 4.0 ± 0.5% air voids. After compaction, specimens were allowed to cool for 24 h and were subsequently cut or conditioned to meet the dimensional requirements for mechanical testing, including ITS, TSR, Cantabro loss, Hamburg wheel tracking, Overlay Test, and SCB fracture energy. The final composition of the studied mixtures is presented in Table 6. It should be noted that RAP aggregate, virgin aggregate, and total binder contents are expressed as wt.% of total mixture mass (Table 6), whereas RAP binder contribution, virgin binder addition, REOW, and SBS contents are expressed relative to the total binder mass (Table 5).

### 2.5. Mixture Testing Methods

A comprehensive mixture-level testing program was conducted to evaluate the mechanical performance of the WMA mixtures incorporating RAP, REOW, and SBS. After compaction and 24-h conditioning at room temperature, specimens were prepared for each performance test following the dimensional and environmental requirements prescribed in the corresponding ASTM and AASHTO standards.

ITS and TSR tests were carried out according to ASTM D6931 [49] and AASHTO T283 [50], respectively. Cylindrical specimens with a diameter of 100 mm and a height of approximately 63.5 mm were tested at 25 °C using a loading rate of 50 mm/min. TSR measurements were obtained by conditioning half of the specimens in a freeze–thaw cycle while keeping the other half dry, allowing the evaluation of moisture susceptibility and adhesive bonding performance in the presence of RAP and rejuvenator.

The raveling resistance of the mixtures was assessed using the Cantabro loss test (ASTM D7064 [51]). This test was performed on 100-mm-diameter gyratory-compacted specimens without steel balls, using 300 drum revolutions at room temperature. The difference between initial and final specimen mass was used to calculate surface abrasion and aggregate retention, providing insight into the cohesion of the modified binder and RAP–virgin aggregate interface.

Rutting performance was evaluated using the Hamburg Wheel Tracking Test (AASHTO T324 [52]). Slab specimens were compacted and cut to the required dimensions before being submerged in a 50 °C water bath (see Figure 3b). The wheel tracking device applied repeated loading cycles up to 20,000 passes while monitoring rut depth progression. The test allowed identification of permanent deformation behavior, rutting rate, and the occurrence of stripping inflection points in the presence of REOW and SBS.

Cracking resistance was examined using both the OT and the SCB test. The OT was performed following Tex-248-F [53] to assess reflective cracking susceptibility under repeated opening–closing displacements. Beam specimens were conditioned at 25 °C and subjected to cyclic displacements of 0.025 inches, from which normalized peak load degradation and stripping inflection patterns were obtained. The SCB test was conducted in accordance with ASTM D8044 [54] at 0 °C using semicircular specimens with a 50-mm thickness and a 15-mm notch depth (see Figure 3c). Load–displacement curves were used to determine fracture energy and post-peak softening behavior, enabling evaluation of low-temperature cracking performance.

## 3. Results and Discussions

### 3.1. Binder Testing Results

All results presented in this section are discussed relative to the control binder (B0), which serves as the unmodified reference binder. In this research, the primary objective of modifying the 70/30 virgin–RAP binder was to restore the excessively stiff and embrittled characteristics of the recovered PMB while achieving a balanced rheological profile suitable for warm-mix asphalt production. REOW was incorporated to reduce stiffness, improve workability, and partially compensate for the loss of light fractions due to aging, thereby lowering softening temperature and enhancing fatigue resistance. SBS was introduced to rebuild the elastic polymer network and improve high-temperature rutting resistance and aging durability. From an environmental standpoint, the combined approach also supports waste reuse by valorizing engine oil waste and maximizing RAP utilization. Together, these objectives aimed to obtain a modified binder system with performance characteristics comparable to or better than conventional PMB grades while enabling sustainable WMA production using 30 wt.% RAP.

#### 3.1.1. Basic Rheological Properties

In this study, binder workability is assessed indirectly through rotational viscosity measurements at 135 °C, which are widely used as indicators of mixing and compaction feasibility in warm-mix asphalt systems.

**Figure 4:**
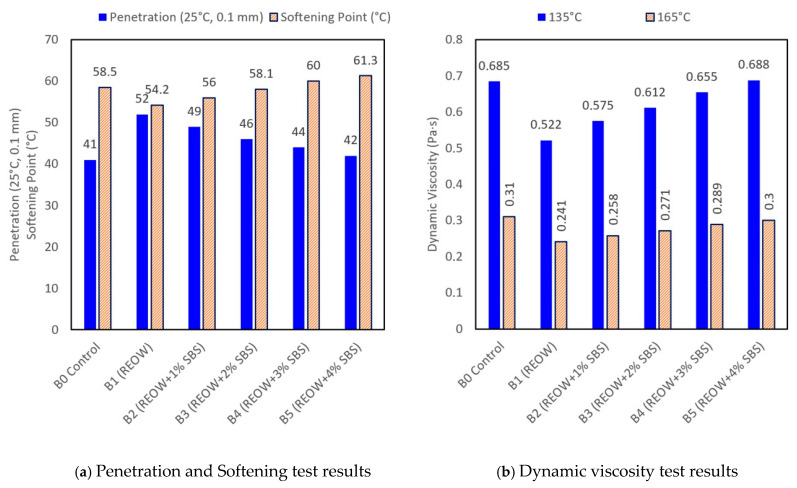
Basic rheological properties of biner.

The conventional binder properties (penetration, softening point, and rotational viscosity) showed clear trends associated with REOW softening and incremental SBS modification (Figure 4). The penetration results demonstrated that the REOW-only binder (B1) became noticeably softer than the control B0, increasing from 41 to 52 dmm, consistent with the rejuvenator’s ability to restore maltene fractions and reduce RAP binder stiffness. As SBS content increased from 1 wt.% to 4 wt.% (B2–B5), penetration decreased progressively from 49 to 42 dmm, reflecting the formation of a stiffer polymer-modified network. This stiffness increase was also evident in the softening point, which rose from 54.2 °C (B1) to 61.3 °C (B5), while the control binder exhibited an intermediate value of 58.5 °C due to the presence of aged PMB from the RAP fraction. Although several rheological parameters increased approximately linearly with SBS content, the selected formulation corresponds to the point at which all key performance criteria are simultaneously satisfied without exceeding practical viscosity limits.

The designation of a “balanced” response is based on satisfying multiple quantitative performance criteria rather than on relative comparison alone. Adequate stiffness was defined as achieving a softening point of 56–60 °C, MSCR Jnr_3.2_ ≤ 1.5 kPa^−1^, and viscosity at 135 °C within 1.6–2.2 Pa·s, which collectively indicate sufficient rutting resistance while maintaining acceptable mixing and compaction characteristics, as indicated by rotational viscosity values at 135 °C within the recommended range for warm-mix asphalt production. Excessive hardening was identified when further SBS addition resulted in viscosity exceeding 2.5 Pa·s and only marginal improvement in MSCR recovery, offering limited additional performance benefit.

Viscosity measurements at 135 °C and 165 °C further confirmed the softening effect of REOW on the blended binder systems. Compared with the control binder, the REOW-modified binder exhibited a reduction in rotational viscosity from 0.685 to 0.522 Pa·s at 135 °C and from 0.310 to 0.241 Pa·s at 165 °C. This reduction reflects the plasticizing influence of REOW on the aged binder fraction, while subsequent SBS addition resulted in a gradual viscosity increase associated with polymer swelling and network formation. Notably, all modified binders maintained viscosity levels within commonly accepted handling ranges at both temperatures.

The subsequent increase in viscosity with higher SBS dosages (0.575–0.688 Pa·s at 135 °C) reflects the swelling of SBS chains and increased polymer–binder interactions. Notably, viscosity values at 165 °C remained within typical handling limits for all modified binders, with B4 exhibiting a moderate viscosity rise (0.289 Pa·s) that indicates good processability while retaining polymer-enhanced elasticity. Based on the prior work experience [55], the acceptable handling limits are defined by rotational viscosity values of ≤3.0 Pa·s at 135 °C according to Superpave practice, and preferably within 1.5–2.5 Pa·s for warm-mix asphalt applications to ensure adequate mixing and compaction. These viscosity thresholds are commonly adopted in Superpave binder specifications and WMA practice as indicators of feasible binder handling, pumpability, and aggregate coating during production.

Based on the analysis of physical properties, the binder containing 3 wt.% SBS and 3 wt.% REOW (B4) was selected for subsequent evaluation as a representative modified binder. The trends in softening point and penetration across B1–B4 were incremental, and no distinct rheological differentiation was observed between adjacent dosages (e.g., B3 vs. B4) based on the measured parameters.

Increasing the SBS content to 4 wt.% (B5) resulted in only a marginal increase in softening point relative to B4, while rotational viscosity increased at both 135 °C and 165 °C. These observations indicate that, within the scope of the physical tests conducted, higher SBS dosage primarily influenced viscosity-related characteristics, with limited additional effect on penetration or softening point. Accordingly, B4 was retained for subsequent analyses as the representative formulation for 3 wt.% SBS modification.

#### 3.1.2. MSCR Test Results

**Figure 5:**
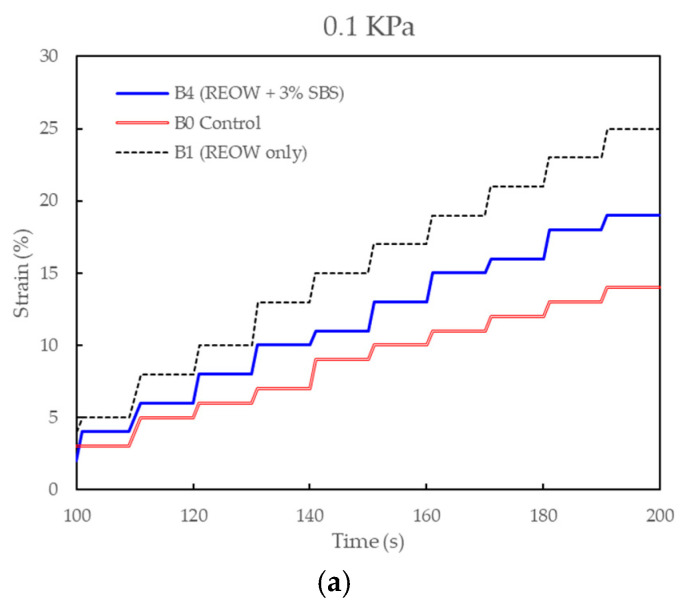

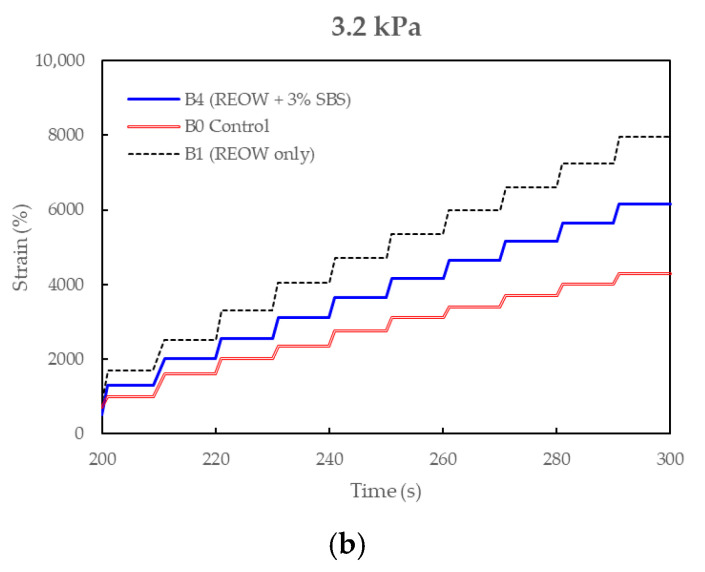
MSCR strain response of RAP–REOW–SBS blended binders at low (**a**) and high (**b**) stress levels.

As shown in Figure 5, the MSCR test was conducted at stress levels of 0.1 and 3.2 kPa to evaluate the stress-dependent viscoelastic and permanent deformation behavior of the investigated binders. Each loading cycle consisted of 1 s creep followed by 9 s recovery, and strain responses are reported as accumulated creep strain and its elastic and non-recoverable components.

Figure 5 illustrates the accumulated strain response over time, while Table 7 summarizes the calculated rheological parameters at stress levels of 0.1 kPa and 3.2 kPa. As shown in Figure 5, the binders exhibited distinct deformation behaviors dictated by their modification state. The control binder (B0) displayed the lowest accumulated strain, reflecting the high stiffness inherent to the aged, oxidized RAP binder.

In contrast, the REOW-modified binder (B1) exhibited the highest accumulated strain (dashed black line), confirming the significant softening effect of the rejuvenator, which increases molecular mobility and reduces resistance to flow. The hybrid binder (B4) showed an intermediate response, indicating that the addition of 3 wt.% SBS effectively reinforces the rejuvenated matrix, restricting deformation compared to the REOW-only system.

Table 7 presents the quantitative MSCR parameters. At the high stress level of 3.2 kPa, the control binder B0 achieved the lowest J_nr_ value of 1.34 kPa^−1^, consistent with the stiff nature of the aged polymer-modified RAP. The addition of REOW in binder B1 notably increased the compliance to 2.48^−1^, demonstrating that the rejuvenator effectively reduces the stiffness of the aged binder.

The hybrid binder B4 achieved a J_nr_ of 1.92 kPa^−1^, indicating that the SBS polymer network partially recovers the stiffness lost during rejuvenation, thereby improving resistance to permanent deformation relative to the REOW-only binder. Regarding elastic response, the control binder maintained the highest percent recovery (R_3.2_ = 58.1%), attributed to the presence of residual, highly aged polymer and the elastic stiffness of the oxidized asphaltenes. The introduction of REOW significantly reduced the recovery to 22.6% in binder B1, suggesting that the rejuvenator dilutes the effective polymer network and promotes viscous flow. However, the hybrid modification in B4 partially restored the elastic recovery to 32.5%. This improvement confirms that the fresh SBS polymer successfully forms a new elastic network within the rejuvenated binder phase, providing a critical enhancement in elasticity that the rejuvenator alone cannot supply.

As shown in Table 8, results from the parametric evaluation indicated that 3 wt.% REOW provided the optimal softening effect without excessive reduction in viscosity, while 3 wt.% SBS resulted in the improvement in elasticity, recovery, and high-temperature performance. Therefore, 3 wt.% was selected as the primary dosage for the B1 (REOW) and B4 (REOW + SBS) binders used in the detailed MSCR testing and subsequent mixture performance evaluation. This selection ensured that the binder systems used in the asphalt mixture tests reflected the most practical and balanced performance within the tested dosage range.

**Table 8:** Key properties of the optimized binder B4 and comparison with typical industry ranges for PMB and WMA application.

Property	Test Standard	B4 (REOW + 3 wt.% SBS)	Typical Industry Range *
Penetration (25 °C, dmm)	ASTM D5 [27]	44	40–60 (PMB/WMA)
Softening point (°C)	ASTM D36 [28]	60.0	≥55 (PMB)
Rotational viscosity @ 135 °C (Pa·s)	ASTM D4402 [30]	0.655	≤3.0 (Superpave); 1.5–2.5 preferred (WMA)
Rotational viscosity @ 165 °C (Pa·s)	ASTM D4402 [30]	0.289	≤0.5–0.7 (handling range)
MSCR Jnr_3.2_ (1/kPa)	AASHTO T350 [56]	1.72	≤2.0 (standard traffic PMB)
MSCR Recovery @ 3.2 kPa (%)	AASHTO T350 [56]	32.1	≥25 (typical PMB)

* Typical ranges compiled from Superpave binder practice, EN 14023 PMB [57] *classes, and published WMA literature* [58].

#### 3.1.3. Intermediate-Temperature Fatigue Parameter (G·sin δ): RAP vs. Modified Binders

**Figure 6:**
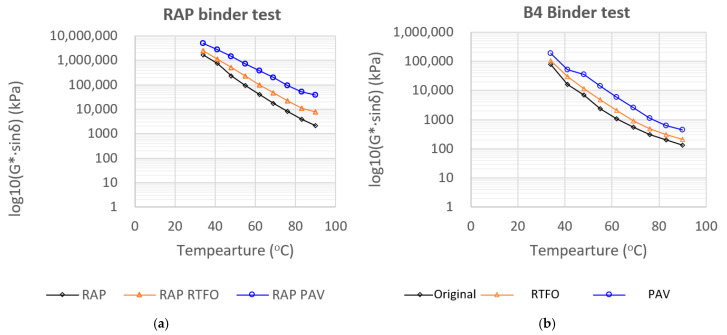
High-Temperature Rutting Parameter of (**a**) Original polymer modified RAP binder vs. (**b**) B4 Binder.

The RAP binder exhibited very high G·sinδ values across the entire temperature range (40–80 °C), confirming its severe aging state and strong susceptibility to fatigue cracking (Figure 6). As the temperature decreased, the RAP binder stiffened rapidly, and both RTFO and PAV aging shifted the curves upward by nearly one order of magnitude, indicating significant loss of viscoelastic damping capacity and extreme brittleness after additional aging. The large vertical separation between the unaged, RTFO, and PAV curves demonstrates that the RAP binder is highly sensitive to oxidative aging, with limited ability to maintain flexibility or resist crack initiation.

In contrast, the B4 hybrid binder (REOW + 3 wt.% SBS) showed substantially lower values and much narrower spacing between aging conditions. The original, RTFO, and PAV curves remained close together, indicating far better resistance to stiffness growth under aging. While the PAV curve still sits above the unaged condition, as expected, the magnitude of the upward shift is small compared with RAP, demonstrating that the combined REOW–SBS system effectively stabilizes the binder against oxidative hardening. Across the entire temperature range, B4 maintained G·sinδ values that were one order of magnitude lower than RAP after PAV aging, confirming its superior intermediate-temperature fatigue performance.

#### 3.1.4. Frequency Sweep Test Results of Binder

**Figure 7:**
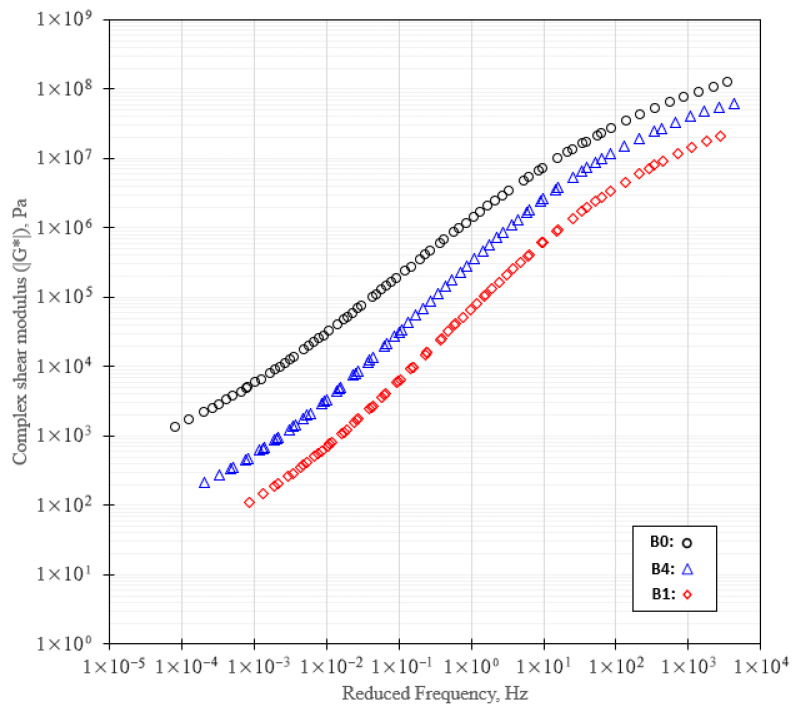
Complex shear modulus (|G*|) from frequency sweep test results.

Figure 7 presents the results of the frequency sweep tests in terms of the complex shear modulus (|G*|) as a function of reduced frequency for the selected binders. Only experimentally obtained data points are shown, and the analysis is restricted to the frequency range supported by the available testing temperatures.

Across the investigated frequency range, all binders exhibit the expected increase in |G*| with increasing reduced frequency, reflecting the transition from viscous-dominated behavior at low frequencies to more elastic-dominated response at higher frequencies. The unmodified binder (B0) consistently shows the highest |G*| over the entire measured range, indicating a comparatively stiffer rheological response.

The incorporation of REOW results in a systematic reduction in |G*|, particularly in the low- to intermediate-frequency domain, which is associated with enhanced binder mobility and a softening effect attributable to the plasticizing action of the oil. This reduction is most pronounced for binder B1, where REOW is present without polymer modification.

At the highest SBS dosage (B4), the binder exhibits |G*| levels approaching those of the unmodified binder in the high-frequency region, while maintaining lower stiffness at low frequencies compared to B0. In this context, the hybrid binder B4 (REOW + 3 wt.% SBS) exhibited an intermediate and favorable rheological response. At low reduced frequencies, B4 showed lower |G*| values than the control binder (B0), indicating that the incorporation of REOW effectively mitigated excessive stiffness. With increasing reduced frequency, the |G*| values of B4 gradually approached those of B0, reflecting the contribution of SBS to stiffness enhancement under short-term loading.

### 3.2. Asphalt Mixture Test Results

#### 3.2.1. Moisture-Conditioned ITS and TSR Results

**Figure 8:**
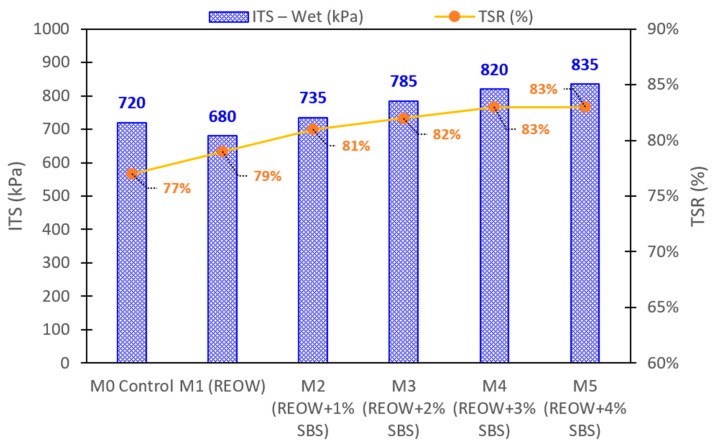
Summary of ITS values and TSR for moisture-conditioned asphalt concrete mixtures.

The moisture-conditioned ITS results and the corresponding TSR values are summarized in Figure 8 and reveal clear trends associated with REOW rejuvenation and SBS modification. The control mixture M0, containing 30 wt.% RAP and no additives, exhibited a wet ITS of 720 kPa and a TSR of 77%, reflecting the inherently brittle and oxidation-hardened nature of the RAP binder. When 3% REOW was incorporated (M1), the wet ITS slightly decreased to 680 kPa, yet the TSR improved to 79%. This modest increase in moisture resistance is consistent with the rejuvenator’s ability to restore maltene fractions and reduce stiffness gradients between virgin and RAP binders, leading to more uniform interfacial bonding within the mixture.

The addition of SBS yielded progressive improvements in both wet ITS and TSR. At 1% SBS (M2), wet ITS increased to 735 kPa with a TSR of 81%, surpassing both M0 and M1. As SBS dosage increased to 2% and 3 wt.% (M3 and M4), wet ITS rose to 785 and 820 kPa, respectively, while TSR improved to 82–83%. These gains demonstrate that SBS improves moisture resistance by enhancing the cohesive strength of the modified binder and reducing moisture-induced debonding at aggregate–binder interfaces. The highest performance was observed in M5, where 4 wt.% SBS resulted in an 835 kPa wet ITS and a TSR of 83%, indicating that the polymer network developed at this dosage effectively counteracts moisture susceptibility without compromising flexibility.

#### 3.2.2. Cantabro Loss

**Figure 9:**
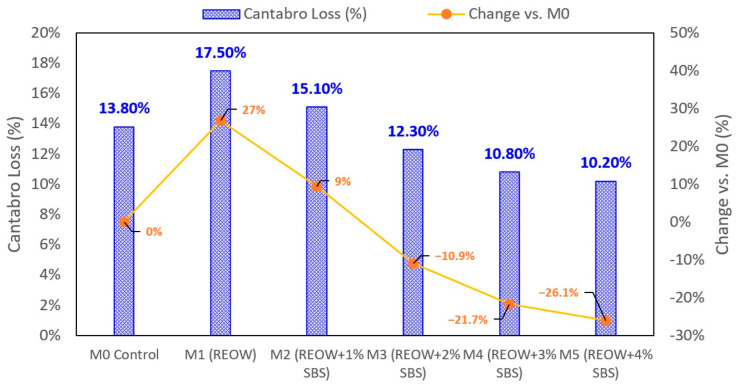
Cantabro test results.

The Cantabro test results presented in Figure 9 reveal clear differences in raveling resistance among the mixtures and highlight the complementary influence of REOW and SBS on mixture cohesion. The control mixture M0 exhibited a Cantabro loss of 13.8%, reflecting the moderate cohesion typically associated with mixtures containing 30 wt.% RAP and a stiff, aged binder. Incorporation of REOW in mixture M1 increased the Cantabro loss to 17.5%, corresponding to a 26.8% increase relative to the control. In this case, excessive over-softening in M1 (REOW-only) may lead to unstable binder films, increased binder drainage, and non-uniform aggregate coating, particularly in mixtures containing RAP aggregates that are already partially coated with aged binder.

As SBS was added in mixtures M2–M5, the Cantabro loss decreased progressively, confirming the reinforcing role of the polymer network. At 1 wt.% SBS (M2), the loss dropped to 15.1%, indicating partial recovery of cohesion as SBS improves binder elasticity and adhesion. At 2 wt.% SBS (M3), the loss decreased to 12.3%, which is 10.9% lower than the control and marks a transition from softened to reinforced mixture behavior. The best performance was observed in M4 and M5, where SBS dosages of 3–4% reduced Cantabro loss to 10.8–10.2%, corresponding to 20–26% improvements relative to M0. These reductions demonstrate that SBS effectively enhances film strength, increases binder–aggregate bonding, and improves the mixture’s resistance to raveling under abrasion.

#### 3.2.3. Overlay Test Results

**Figure 10:**
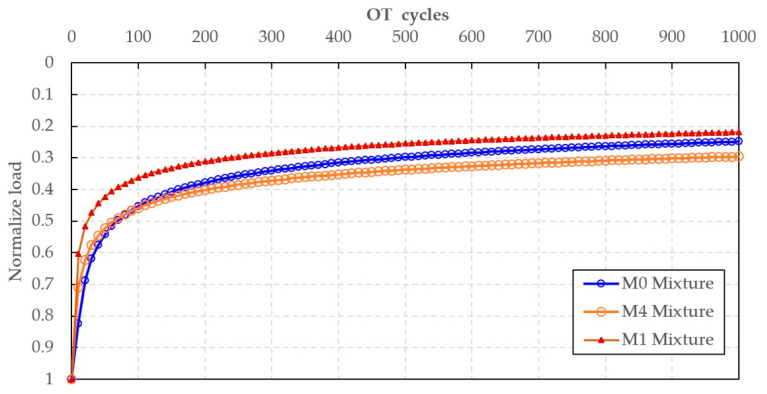
OT test results.

The Overlay Test results for mixtures M0, M1, and M4 demonstrate clear differences in crack resistance that reflect the combined effects of RAP aging, REOW rejuvenation, and SBS polymer modification (Figure 10). The M1 mixture, produced with REOW-modified binder and no SBS, exhibited the most rapid loss of normalized load, decreasing sharply during the first 100 cycles and approaching a final normalized load of approximately 0.21 at 1000 cycles. This rapid degradation indicates poor crack resistance, consistent with the softer, more viscous rheology induced by REOW, which reduces cohesive strength and accelerates crack propagation under repeated opening–closing cycles.

In contrast, the control mixture M0 displayed intermediate cracking performance, with a slower decline in normalized load and a final residual load of about 0.25. Although the aged PMRB in RAP increases mixture stiffness, its brittle nature limits its ability to resist fatigue-induced cracking. The curve shape reflects this behavior: a pronounced initial drop due to low tensile flexibility, followed by a gradual stabilization as fracture resistance transitions from binder-controlled to aggregate-interlock-controlled behavior. The performance ranking of M0 between M1 and M4 is consistent with earlier ITS, TSR, and Cantabro results, where M0 showed relatively high stiffness but limited ductility.

The hybrid mixture M4, incorporating both REOW and 3 wt.% SBS achieved the highest fatigue resistance of all mixtures tested. Its normalized load decreased more gradually throughout the test, and the residual load at 1000 cycles (~0.30–0.32) was significantly higher than that of M0 and M1. This improved cracking performance is attributed to the formation of a continuous SBS polymer network that enhances elastic recovery, dissipates crack tip stresses, and delays microcrack growth. At the same time, the presence of REOW maintains adequate flexibility, preventing the mixture from becoming overly brittle.

#### 3.2.4. SCB Test Results and Fracture Energy Evaluation

**Figure 11:**
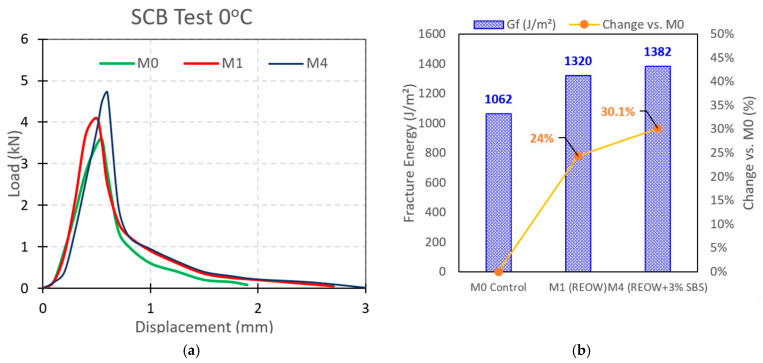
(**a**) SCB Test results and (**b**) fracture energy calculation results.

The SCB results at 0 °C show clear differences in low-temperature cracking resistance among the three mixtures, highlighting the combined effects of RAP aging, REOW rejuvenation, and SBS reinforcement. Figure 11a presents the load–displacement responses, and Figure 11b summarizes the corresponding fracture energy values $G_{f}$ calculated from the measured work of fracture *W_f_* normalized by the ligament area $A_{\mathrm{lig}}$. The overall findings are presented in Table 9.

The control mixture (M0) exhibited the lowest fracture energy (1062 J/m^2^), reflecting the brittle behavior expected from an aged RAP binder system. Although M0 developed a moderate peak load (3.58 kN at 0.55 mm), the load decreased rapidly after peak, indicating limited post-peak crack-opening capacity and fast propagation of the notch-initiated fracture.

The REOW-modified mixture (M1) showed an improved fracture profile. M1 reached a slightly higher peak load than M0 (4.10 kN at 0.50 mm) and exhibited a more gradual post-peak softening with a longer displacement tail. Consequently, the work of fracture increased (2.31 J vs. 1.86 J for M0), leading to a higher fracture energy of 1320 J/m^2^, which corresponds to an improvement of +24.3% relative to the control. This improvement may be attributed to REOW’s rejuvenating action on the aged RAP binder, which restores maltene-like fractions, enhances binder flexibility, and delays crack propagation, thereby allowing greater crack opening before complete failure.

The best overall cracking resistance was achieved by the hybrid mixture (M4) containing REOW and 3 wt.% SBS. M4 produced the highest peak load (4.70 kN at 0.60 mm) and maintained the slowest post-peak reduction, resulting in the largest work of fracture (2.42 J) and the highest fracture energy (1382 J/m^2^). This corresponds to an improvement of +30.1% over M0 and approximately +4.7% over M1. The enhanced behavior is attributed to SBS-induced polymer network reinforcement, which increases crack-tip stress redistribution and improves resistance to unstable crack growth, while REOW mitigates excessive stiffness that could otherwise promote brittle fracture. Overall, the combined REOW–SBS modification provides a favorable balance between strength and ductility, enabling M4 to sustain greater crack growth prior to failure.

#### 3.2.5. The Hamburg Test Result

**Figure 12:**
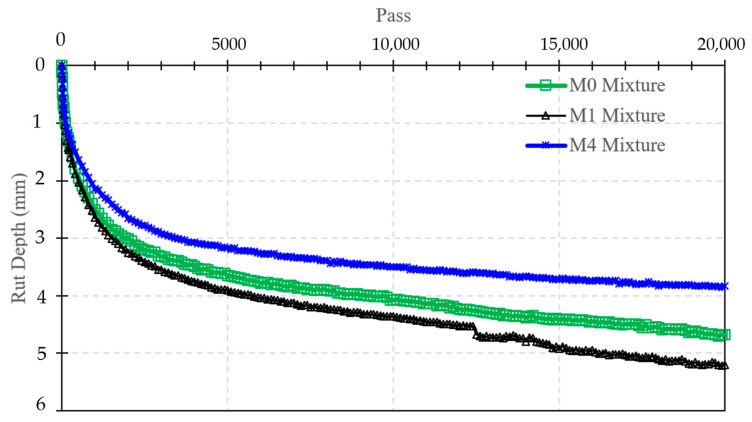
Hamburg Wheel Tracking test results.

The Hamburg wheel tracking results confirm that the REOW–SBS hybrid mixture (M4) provides the best rutting resistance, with the control mixture (M0) performing moderately and the REOW-only mixture (M1) showing the highest rut susceptibility. As shown in Figure 12, during the initial consolidation phase (first ~1000 passes), rut depths remained below about 1.6 mm for M4, 1.8 mm for M0, and 2.0 mm for M1, already indicating a stiffer, more rut-resistant response for the SBS-modified mixture. By 5000 passes, the rut depths had increased to roughly 2.7 mm (M4), 3.0 mm (M0), and 3.3 mm (M1); thus, M4 carried about 10% less rutting than M0 and ~18% less than M1 at this intermediate traffic level. At 10,000 passes, the gap widened, with rut depths of approximately 3.4 mm (M4), 3.8 mm (M0), and 4.2 mm (M1), showing that the REOW-only mixture accumulated about 24% more rutting than the SBS-modified mixture. By the end of the test at 20,000 passes, M4 still limited rut depth to around 3.9 mm, compared with 4.4 mm for M0 and 4.9 mm for M1, meaning the hybrid modification reduced permanent deformation by roughly 12% relative to the control and 20% relative to the REOW-only mixture.

A clear stripping inflection was observed only for M1, with a noticeable increase in rutting rate beginning at around 12,400 passes, indicative of moisture-induced damage and loss of aggregate–binder adhesion in the softened REOW mixture. In contrast, both M0 and M4 maintained nearly linear, stable rutting trends without a distinct stripping phase up to 20,000 passes, suggesting that the aged RAP binder (M0) and especially the SBS-reinforced binder (M4) provide better resistance to moisture-rutting interaction. These Hamburg results are consistent with the MSCR, TSR, and Cantabro findings, which showed that REOW alone improves flexibility but weakens high-temperature performance, whereas the REOW + 3 wt.% SBS system in M4 effectively restores rutting resistance while preserving adequate workability.

Table 10 compares the measured asphalt mixture performance indicators with commonly adopted specification criteria or guidance values reported in standards and field practice. Moisture resistance is evaluated using the ≥80% TSR criterion associated with AASHTO T283, durability and rutting resistance are contextualized using Cantabro and Hamburg limits commonly applied in practice, and cracking-related indicators (ITS and SCB fracture energy) are compared with guidance ranges reported in related studies. This comparison shows that the REOW–SBS hybrid mixture (M4) provides the most balanced performance and generally falls within commonly accepted ranges for RAP-containing warm-mix asphalt, while acknowledging that final acceptance depends on agency- and project-specific specifications.

## 4. Conclusions

This study investigated the feasibility of combining recycled engine oil waste and SBS polymer to rehabilitate polymer-modified RAP binder and to produce high-RAP warm-mix asphalt containing 30 wt.% RAP. The following conclusions can be drawn:

Binder-level performance recovery was quantitatively confirmed. The recovered RAP binder exhibited severe aging, whereas hybrid modification using 3 wt.% REOW and 3 wt.% SBS (B4) restored rheological profile. Compared with the control binder, B4 achieved a penetration of 44 dmm and a softening point of 60.0 °C, while maintaining rotational viscosities of 0.655 Pa·s at 135 °C and 0.289 Pa·s at 165 °C. MSCR results showed that REOW increased non-recoverable creep compliance at 3.2 kPa from 1.34 to 2.48 kPa^−1^ while reducing recovery from 58.1% to 22.6%, whereas SBS incorporation moderated this effect, yielding a response with Jnr of 1.92 kPa^−1^ and recovery of 32.5%.

Mixture performance results demonstrate clear benefits of the REOW–SBS hybrid system. At the mixture scale, the hybrid mixture (M4) achieved a wet ITS of 820 kPa and a TSR of 83%, exceeding the commonly adopted 80% moisture resistance criterion, whereas the control mixture remained below this threshold (77%). Cantabro loss was reduced from 13.8% (M0) to 10.8% (M4), indicating improved resistance to raveling. Hamburg wheel tracking results showed that rut depth at 20,000 cycles decreased from 4.4 mm (M0) and 4.9 mm (M1) to 3.9 mm for M4, corresponding to an approximately 12–20% reduction in permanent deformation.

Cracking resistance was markedly enhanced through the combined effects of REOW rejuvenation and SBS polymer reinforcement. The SCB fracture energy increased from 1062 J/m^2^ for the control mixture (M0) to 1320 J/m^2^ with REOW modification alone (M1) and further to 1382 J/m^2^ for the REOW–SBS hybrid mixture (M4), corresponding to improvements of approximately 24% and 30%, respectively, relative to the control. Overlay Test results showed a higher residual load at 1000 cycles (≈0.30–0.32 for M4 versus ≈0.25 for M0), confirming the superior resistance of the hybrid mixture to fatigue-induced cracking.

Overall, the hybrid REOW–SBS mixture (M4) delivered the most balanced performance, achieving 14% higher wet ITS and 6% higher TSR than the control, 22–26% lower Cantabro loss, and approximately 20% lower Hamburg rut depth after 20,000 passes without evidence of stripping. It also produced about 30% higher SCB fracture energy and the highest Overlay Test residual load, demonstrating that the combination of REOW-induced flexibility and SBS-derived elasticity acts synergistically to resist cracking, raveling, and permanent deformation in RAP-rich warm-mix asphalt systems.

The findings are based on laboratory-scale testing using a single RAP source derived from a polymer-modified surface course and a fixed RAP content of 30 wt.%. Long-term field validation, evaluation under different RAP sources and polymer types, and durability under extended aging conditions were not addressed in this study. Therefore, while the results demonstrate technical feasibility and performance potential, direct field implementation should be preceded by project-specific mixture design verification and agency acceptance testing.

## Figures and Tables

**Table 5:** Binder composition (binder-level, wt.% of total binder mass).

Binder ID	RAP Binder Contribution (%)	Virgin Binder Added (%)	REOW (wt.% of Binder)	SBS (wt.% of Binder)
B0	30	70	0.0	0.0
B1	30	70	3.0	0.0
B2	30	70	3.0	1.0
B3	30	70	3.0	2.0
B4	30	70	3.0	3.0
B5	30	70	3.0	4.0

*Note: RAP binder contribution and virgin binder addition sum to 100*% *of the base binder blend. REOW and SBS contents are expressed as* wt.% *relative to the total binder mass.*

**Table 6:** Asphalt concrete composition (mixture-level, wt.% of total mixture mass).

Mix ID	RAP Aggregate	Virgin Aggregate	RAP Binder Contribution	Virgin Binder Added	Total Binder
M0	30	70	1.6	3.4	5.0
M1	30	70	1.6	3.4	5.0
M2	30	70	1.6	3.4	5.0
M3	30	70	1.6	3.4	5.0
M4	30	70	1.6	3.4	5.0
M5	30	70	1.6	3.4	5.0

**Table 7:** MSCR parameters at 0.1 and 3.2 kPa.

Binder	σ (kPa)	ε_peak_ (%)	ε_e_ (%)	ε_ρ_ (%)	Jnr (kPa^−1^)	% Recovery (R)
B0—Control	0.1	25	11	14	0.14	44.0
	3.2	4300	2500	1800	1.34	58.1
B1—REOW only	0.1	35	10	25	0.25	28.5
	3.2	7950	1800	6150	2.48	22.6
B4—Hybrid	0.1	30	11	19	0.19	36.6
	3.2	6150	2000	4150	1.92	32.5

**Table 9:** Summary of SCB fracture parameters at 0 °C.

Parameter	Unit	M0 (Control)	M1 (REOW)	M4 (REOW + 3% SBS)
Peak load	kN	3.58	4.10	4.70
Displacement at peak load	mm	0.55	0.50	0.60
Work of fracture, $\left(W_{f}^{*}\right)$	J	1.86	2.31	2.42
Ligament area, $\left(A_{\mathrm{lig}}\right)$	m^2^	0.00175	0.00175	0.00175
Fracture energy, $\left(G_{f}=W_{f}/A_{\mathrm{lig}}\right)$	J/m^2^	1062	1320	1382
Improvement vs. M0	%	-	+24.3	+30.1

* The work of fracture W_f_ was calculated as the area under the load–load-displacement curve using trapezoidal numerical integration. Load and displacement were recorded in kN *and* mm*, respectively; therefore, 1 kN mm = 1 J . The ligament area was calculated as $A_{lig}=t\left(R-a\right)$, where the specimen thickness t = 50* mm*, the specimen radius R = 50* mm*, and the notch depth a = 15* mm*.*

**Table 10:** Asphalt mixture performance results and reference specification or guidance.

Performance Indicator	Typical Specification/Guidance Range	Reference Standard or Guidance	Control (M0)	REOW (M1)	REOW–SBS Hybrid (M4)
Indirect Tensile Strength, ITS (kPa)	≥700	Suggestion from related work and field construction experience [59,60,61,62]	720	680	820
Tensile Strength Ratio, TSR (%)	≥80	AASHTO T283 (agency-adopted criterion) [50]	77	79	83
Cantabro loss (%)	≤20 (dense-graded mixtures)	Common agency practice; ASTM D7064 used for test [51]	13.8	17.5	10.8
Hamburg rut depth (mm @ 20,000 cycles)	≤12.5	AASHTO T324 (limit set by agency specification) [52]	4.4	4.9	3.9
SCB fracture energy (J/m^2^)	≥500	Suggestion from related work and field construction experience [59,60,61,62]	930	1150	1210

## Data Availability

The data presented in this study are available within the manuscript. Additional data related to this work are available from the corresponding author upon reasonable request.

## Abbreviations

List of abbreviations used in this study

Abbreviation	Definition
DSR	Dynamic Shear Rheometer
G*·sinδ	Fatigue-related rheological parameter
ITS	Indirect Tensile Strength
Jnr	Non-recoverable creep compliance
MSCR	Multiple Stress Creep Recovery test
OT	Overlay Test
PMB	Polymer-Modified Bitumen
RAP	Reclaimed Asphalt Pavement
REOW	Recycled Engine Oil Waste
R%	Percent recovery
SBS	Styrene–Butadiene–Styrene
SCB	Semi-Circular Bending test
TSR	Tensile Strength Ratio
WMA	Warm-Mix Asphalt
WTT	Hamburg Wheel-Tracking Test
